# A Behind-the-Scenes Story of Precision Medicine^*^

**DOI:** 10.1016/j.gpb.2017.01.002

**Published:** 2017-02-07

**Authors:** Maynard V. Olson

**Affiliations:** Department of Genome Sciences and Department of Medicine, University of Washington, Seattle, WA 98195, USA

Let me start with a few words about the goals and intended audience for this introduction to republication of two reports of the U.S. National Research Council (NRC) – *Mapping and Sequencing the Human Genome* (1988) and *Toward Precision Medicine* (2011). I do not intend to summarize what the reports say. They are both short, well written, and can speak for themselves. I encourage readers who want a quick summary to read these reports the way most policy-makers do: start with the Executive Summaries, particularly the Conclusions and Recommendations. NRC committees write these summaries with great care since they are read far more widely, and by a more influential audience, than the bodies of the reports. With respect to audience, I assume this book will be of greatest interest to scientists and policy-makers interested in how the policy-making apparatus in the United States works and how it shaped two key initiatives in biology and medicine during their formative stages.

A scientist myself, now retired from active research, I am the only person who served on both the Mapping and Sequencing and Precision Medicine Committees. My active research career happened to span the remarkable quarter century during which we sequenced the first human genome and learned enough about it to be ready to launch a major effort to apply this knowledge to mainstream medicine. Because of this fortuitous timing, I was able to serve as one of the most junior members of the Mapping and Sequencing Committee and one of the most senior members of the Precision Medicine Committee.

What I hope to add to this republication is an insider’s view of the way these committees came into existence and how they functioned. It is one scientist’s view. There are many stakeholders in the science-policy process: scientists, agency administrators, Congressional staff, politicians, special interest groups, and the general public. However, a hallmark of the NRC process is that active scientists volunteer their time to participate in policy-making at a formative level. It is an exemplary process that has proven itself repeatedly across the whole range of scientific and technical issues that arise in modern economies and political systems. I think the involvement of active scientists in the initial stages of policy-making is the most important reason the system has been so successful. There are other reasons, relating to the culture of NRC deliberations: the committees are carefully selected after extensive consultation with knowledgeable advisers; they deliberately include scientists known to differ in their attitudes toward, opinions about, and experience with the subjects under study; it is a consensus process that involves extensive face-to-face discussion among people who respect each other even though they often disagree. The risk of emphasizing consensus as strongly as the NRC process does is that committees will only be able to agree on a bland set of recommendations. However, time and again, NRC committees make bold, innovative recommendations that influence subsequent developments. My hope is that the mixture of historical perspective, anecdote, and considered opinion I offer in this Introduction will encourage scientists and policy makers to consider how the NRC model might help them confront the policy challenges they face.

The Mapping and Sequencing Committee met a long time ago, before many young scientists working in genomics today were born. Hence, a little background about that era may help put the *Mapping and Sequencing* report in historical context. When I started studying the yeast genome, my only tools were the restriction enzyme *Eco*RI and agarose-gel electrophoresis. I remember the first time, in 1974, when I saw an ethidium-bromide-stained gel of yeast DNA cleaved with *Eco*RI. It remains one of the most exciting moments of my career. This digest produces thousands of fragments so there were not discrete bands, at least from the single-copy DNA that comprises most of the genome. Nonetheless, the gel was not just a blur: it looked just like it should have if the yeast genome contained a few thousand randomly positioned *Eco*RI sites, each of them in the same position in every copy of the genome. Right away, I wanted to map these sites and put the genes on the map. I had no idea how to do this but could see that it was doable. I was reminded of having read John Kendrew’s description of the first time he saw an X-ray diffraction pattern of a hemoglobin crystal. He could see that the molecules in the crystal all had the same structure and the crystal was well ordered; hence, he knew it should be possible to figure out the positions of all the atoms. Of course, the figuring-out part took 25 years [1]. Mapping the yeast genome was not that hard a problem, but it did take 10 years from the time I started serious work in 1979 before I had a reasonably complete map.

Oddly, during this whole period I had little competition despite the existence of a rapidly expanding community of yeast researchers, most of whom were studying their own little parts of the genome. The only comparable initiative was John Sulston’s and Alan Coulson’s project to map the nematode genome. Every now and then, John, Alan, and I would meet at a pub near Cambridge, drink a few pints of English bitter, and compare notes. We had a friendly competition, as evidenced by our decision to publish our first papers on our projects back-to-back in the *Proceedings of the National Academy of Science* (PNAS) in 1986 [2], [3]. Sydney Brenner communicated both of them to the PNAS.

Why was there so little activity in genomics during this period? There are many reasons, of which I will emphasize only two. The first stems from the history of molecular biology. From 1953 onward, as molecular biology emerged as a well-defined field following the discovery of the double helical structure of DNA, its practitioners focused overwhelmingly on mechanism. How does DNA replicate? How are proteins synthesized? How is gene expression regulated? Molecular biologists developed first-order answers to these questions in remarkably short order. They did so through cult-like allegiance to the hypothetico-deductive method. Small research teams, even individuals, framed, tested, and refined hypotheses. Despite the primitive experimental methods available, they got answers in days, weeks, or, at most, months. If they became stuck, they modified their goals. There was a lot to do, and time was of the essence. By asking specific questions, making good choices of experimental system, and designing decisive experiments, they made rapid progress. Not much science actually works this way, but molecular biology did during its glory days.

With the advent of recombinant-DNA techniques in the early 1970’s there was an explosion of work of this type. All genes in all organisms suddenly became accessible to study. New processes such as splicing and RNA editing came into view. Attention shifted from the mechanisms of relatively simple processes, such as the regulation of the *lac* operon in *Escherichia coli*, to more complex ones. However, the research paradigm did not budge. There seemed no reason to tamper with success. New techniques were certainly welcome, but molecular biologists felt they had discovered the all-time secret to making rapid scientific progress. There was no room for genome projects in this space.

A second reason genomics got off to a slow start was philosophical. The divide-and-conquer strategy of molecular biology’s early years was thoroughly reductionist. The key to understanding cells was to take them apart, identify their molecular components, and then study how these components interact, a few at a time, to produce functional effects. The notion was that the sum of all these effects would provide a complete picture of how cells and organisms work. That is the reductionist’s creed. I was skeptical of it, particularly the notion that what molecular biology needed was more mechanistic detail about individual instances of cellular processes such as transcription, translation, and splicing. My skepticism arose from my training in chemistry. I thought molecular biologists were chemically naïve. In my view, there was no reason to think a piecemeal approach to cells would ever converge. To me, the chemical mess looked impenetrably complex.

It was none other than Jim Watson who changed my mind. Although I later came to know Jim well during my work on the Human Genome Project, Watson’s biggest influence on me occurred years earlier through his authorship of *The Molecular Biology of the Gene* [4]. In the early editions of this remarkable textbook, there is a chapter entitled “*A Chemist’s Look at the Bacterial Cell*”. When I read this chapter during the early 1970s, I thought it had been written specifically for me. The chapter gets off to a leisurely start with an outline of the basics of metabolism, descriptions of the ways the small, medium-sized, and large molecules in cells are synthesized, and examples of metabolic pathways. Toward the end of the chapter, Watson sums matters up by drawing a simple metabolic chart. As I read this pretty story, my skepticism mounted page by page. How was Watson ever going to convince me that what he was describing was anything more than the tip of an iceberg. Remarkably, Watson sensed that some of his readers would react in this way and said, as he wrapped up, “It is easy for the sophisticated pure chemist to look at this metabolic chart with initial skepticism.” Quite right! I may not have been sophisticated, but I certainly was skeptical. Watson continued, “The question arises whether this figure, by its simplification, complete misses the point of metabolism in *E. coli*.” Yes! Exactly the right question but how was he going to answer it? He did so brilliantly in a section entitled “*The Significance of a Finite Amount of DNA*”. Using primitive estimates of *E. coli*’s genome size, the average molecular weight of *E. coli* proteins, and the number of steps in typical metabolic pathways, Watson produced an upper bound on the chemical complexity of *E. coli* that still looks good today. I was dumbfounded. He ended the chapter with a flourish:

“Therefore even a cautious chemist, when properly informed, need not look at a bacterial cell as a hopelessly complex object. Instead he might easily adopt an almost joyous enthusiasm, for it is clear that he, unlike his nineteenth-century equivalent, at last possesses the tools to describe completely the essential features of life.”

The genome was the key: it places the only defensible constraint on biological complexity. I was hooked.

I will not recount my struggles from 1979–1989 to map the restriction sites across the yeast genome. It was slow going. I did much of the work myself, assisted by one or two technicians. Available recombinant-DNA tools were woefully inadequate when applied to millions of base pairs. There was no bioinformatics and, of course, no internet or personal computer. I made a deal with a local crystallographer to obtain time-shared access to his mini-computer. We had to run wires through the building’s utility silos to connect my terminal to his computer. When I set up the terminal, the event drew a crowd. “What are you going to do with that”? My colleagues asked, “this is a genetics department”! I worked on the yeast-mapping project for 7 years before publishing my first paper, and this paper only established that what we were doing would probably work if we kept at it.

Meanwhile, the outlandish idea of sequencing the human genome began to create a stir. The chief promoters of the idea, Charles DeLisi and Robert Sinsheimer, were visionaries in leadership positions. I was a junior faculty member at Washington University in St. Louis. Our paths never crossed. Hence, I have nothing to add to published accounts of the critical discussions that occurred during 1985 and 1986 [5], [6]. They culminated in establishment of the NRC Committee on *Mapping and Sequencing the Human Genome*.

The NRC, which is an operational arm of the U.S. National Academies of Science, Engineering, and Medicine, is a cumbersome bureaucracy that produces reports on issues of national interest that have a scientific or technical character. Although slow to act, and characteristically conservative, NRC reports are remarkably authoritative. They get that way because the NRC has a strong staff, recruits outstanding committee members, who serve without pay, respects the autonomy of committees once they are appointed, and subjects reports to a rigorous, multi-stage review process. For the Human Genome study, the NRC recruited Bruce Alberts as chair and appointed a truly remarkable committee. I will not list all the names, which are in the report, but the list includes Jim Watson, Sidney Brenner, Lee Hood, Dan Nathans, and Wally Gilbert. In surveying what relevant work was underway, the staff discovered my little project in St. Louis and invited me to make a presentation to the committee in January, 1987. I was terrified. I had never met any of these people and regarded them with awe. Nonetheless, I gave it my best shot, emphasizing that real progress was being made in scaling up recombinant-DNA technology but that a huge gap remained between the lofty vision of a human-genome sequence and technical reality.

The next month, Wally Gilbert decided to pursue a private-sector approach to genome sequencing and, because of the resultant conflict of interest, resigned from the committee. Shortly thereafter, I received a phone call from John Burris, the Study Director from the NRC staff, who asked me if I would take Gilbert’s place. Now I was in real trouble. It was one thing to show a few slides to this distinguished group and then fly home, another to serve with them and help shape and write what was clearly going to be an epochal science-policy document. Nonetheless, despite my apprehensions, I signed up.

We met several times in 1987 and gradually got to know each other. I was struck by the camaraderie that developed, a tribute to Bruce Alberts’s leadership. The only approximate peer of mine on the committee was Shirley Tilghman, who later became a highly respected President of Princeton University. Although Shirley and I were at about the same stages of our careers, she was much better known than I was and more comfortable around the senior members of the committee. Nonetheless, we had the bond of shared experience at the lab bench. At one coffee break, a discussion started about who on the committee had ever sequenced at least a thousand base pairs of DNA. Shirley and I indisputably had. David Botstein claimed he had, as well, but Shirley and I were dubious. David’s claim was based on having published the sequence of the yeast *URA3* gene, which is only 1170 bp long, and there were two other authors on the paper. It was all good fun. David’s claim was probably true. He did join Shirley and me in injecting a little sobriety into the proceedings. Here we were discussing a project to sequence billions of base pairs of unmapped DNA, and the committee’s collective experience involved sequencing a few thousand base pairs of very well mapped material. Even that experience had been gained at the expense of sweat, tears, and exposure to the β particles ^32^P emits. Fluorescence-based methods were still for the future.

Painstakingly, a draft report emerged. Many committee members had been initially skeptical of the proposal to map and sequence the human genome. Concerns varied. There was much fear that a big project would drain research funding away from more traditional activities. Some members just did not like the idea of giving “big science” a foothold in biology. Others doubted the feasibility of the project or were uncertain, even if it did prove feasible, that the data would be analyzable and useful.

No one line of argument shifted sentiments toward the more favorable consensus we ultimately reached. However, I would cite a few points that I think played important roles:

1. Some senior members of the committee, Dan Nathans comes to mind, understood that we were engaged in a diplomatic negotiation. As in state diplomacy, wording mattered. The phrase “special effort”, which appears in the very first recommendation in the Executive Summary, was important. We recommended that the mapping and sequencing of the human genome “merits a special effort that should be organized and funded specifically for this purpose” – not a “big project” or “crash program”, but a “special effort”. With this phrase, we signaled that we did not think the mapping and sequencing were just going to happen in the normal course of research, but also distanced ourselves from any connotation that we sought a massive disruption of business as usual. Who could be against a “special effort?”
2. We avoided hype. Hype is an easy target for critics and does not help sell initiatives as much as many scientists think it does. Again in the first recommendation, here is what we promised: “a special effort in the next two decades will greatly enhance progress in human biology and medicine.” That is it: no promise to cure cancer, to produce a cornucopia of new drugs, to revolutionize health care – just the promise that project data would “greatly enhance progress in human biology and medicine.”
3. We struck a good balance between arguing that the task was too hard and too easy. Oddly, both positions popped up repeatedly during committee deliberations and during later controversies about implementation of the report. If the job had been portrayed as too hard, the implication would be that a special effort was premature. If portrayed as too easy, it would be that no special effort was needed. I played an active role in this part of the discussion because I had a better feel than most committee members for the strengths and weaknesses of current technology. I knew that a brute-force effort that relied on scaling up 1987 technology would fail. On the other hand, there was a lot of momentum toward developing better techniques so I was optimistic that major improvements in the state of the art would occur by the time large-scale data collection commenced. I recall dictating a statement to this effect over the phone to John Burris – we had no e-mail in those days. The statement appears verbatim in the second recommendation of the Executive Summary: “Although the needed capabilities do not yet exist, the broad outlines of how they could be developed are clear. Prospects are therefore good that the required advanced DNA technologies would emerge from a focused effort that emphasizes pilot projects and technological development.”
4. We argued for including model organisms. Oddly, this was a novel policy idea – early discussions had focused entirely on the human genome. We were blunt in stating that “To succeed, … this project must not be restricted to the human genome; rather it must include an extensive sequence analysis of the genomes of selected other species.” We called out bacteria, yeast, *Drosophila melanogaster*, and *Caenorhabditis elegans* for special emphasis. I was a vigorous supporter of this position. My scientific roots were in the yeast community; hence, I knew firsthand that much of the vigor and vitality of biological research was embedded in organism-specific research communities. These communities developed techniques, concepts, and research problems that were characteristically more holistic than those applicable to all organisms. The communities had their own value systems and were powerful social networks. If genomics was good for human biology, it would certainly be good for the model organisms. Also, the collective size of the genomes of the most attractive genome-sequencing targets among model organisms was less than ten percent that of the human genome. The genome project was not going to succeed or fail because it took on this extra challenge. The inclusion of model organisms greatly increased support for the project within the scientific community and, as it developed, played a key role in attracting top-tier scientists to it. For example, it is doubtful that Bob Waterston and John Sulston would have made major commitments to mapping and sequencing the human genome if they had not been able to sequence the genome of their first love, *C. elegans*, during the early years of the Human Genome Project. A more subtle point relating to model organisms is that we left the title of the report *Mapping and Sequencing the Human Genome* despite our strong recommendation that the project include model organisms. In this way, we made model-organism sequencing an essential part of the Human Genome Project rather than an add-on. They were part of the main project, not something else we wanted to see happen. Many of our colleagues in model-organism communities missed these subtleties and opposed the Human Genome Project out of short-sightedness and political naïvité. Nonetheless, they benefited tremendously from our strategy. Before the Human Genome Project, it was always difficult to explain within the political system why research on yeast was important. After the project, the tight linkage of yeast research to medical research was secured. The point here is not that it is impossible to justify yeast research in its own right: it is that the reasons are difficult to explain during inevitable competitions for limited resources.
5. We got the time scale and budget right. I have told the story about how this happened before, but it is still not well known [7]. We estimated that targeted funding of $200 million/yr would be required for the Human Genome Project for 15 years, a total of $3 billion/yr. In standard accounts, this figure was interpreted as reflecting an estimated cost of $1/bp. Actually, we had no idea what the cost per base pair would be since we were counting on technology that had not yet been invented to get the job done. The decisive voice on budget was the committee member, Jim Watson, who perhaps knew the least about the nuts-and-bolts issues that would ultimately determine costs. Watson justified the 15-year duration for the project and the $200 M/yr annual costs with an argument that had nothing to do with projected costs of facilities, labor, equipment, and supplies. He advocated 15 years as a compromise between 10 and 20 years. The problem with a 10-year project, he argued, is that one could barely get a 10-year project off the ground before critics would begin to say it was falling behind schedule. However, a 20-year project would never sell because institutions do not plan on such long time scales: if one asks support for a 20-year project, the most likely response is that the whole idea is premature. So, 15 years it would be. The $200 M/yr figure related to the political system in the United States. I still remember the confused silence when Watson made the following pronouncement: “The budget should be $200 M/yr; anything bigger would be a fat target during annual budget reviews, while with anything smaller there will not be anything in Illinois.” The first point made sense, but what did Illinois have to do with the estimated cost of sequencing the human genome? Watson, as usual, was a few steps ahead of the rest of us. His point was that the project would have more political support if it were distributed around the United States. Roughly one third of the states, including Illinois, might plausibly compete to host potions of a project of this type. If the budget were much less than $200 M/yr, there would not be enough money to fund activities in many of them, particularly since a few big states with multiple, strong research centers would inevitably soak up most of the money. A project whose only sites were in California, Massachusetts, and New York, for example, would have less political support than one that included Illinois – and, by implication, a number of other states with similar concentrations of academic activity.

Some will argue that the above list puts too much emphasis on the diplomatic and political dimensions of the science-policy process. The *Mapping and Sequencing* report does do a credible job, given the state of molecular biology in the late nineteen eighties, of explaining the rationale for sequencing the human genome and the steps that would have to be taken to get the job done. This was essential to the report’s credibility and is the hallmark of the NRC process. Nonetheless, the technical details in a report of this type, essential as they are, have less influence than the report’s overall message. Diplomatic and political nuances largely determine that message.

With respect to things we got wrong, the outstanding example is our treatment of intellectual property. We underestimated how big a threat gene-patenting would pose to the scientific culture of genomics. We devoted a single paragraph to the topic in the last few pages of the report in a chapter entitled “*Implications for Society*”. In that paragraph, we posed some of the right basic questions, recommended that the issues “be given prompt study by an independent body” and expressed our view that “genome sequences should be a public trust and therefore should not be subject to copyright”. However, we focused too much on copyrights and not enough on patents. More seriously, we buried the discussion so deeply that our brief comments had no influence on subsequent events. The NRC process typically handles scientific, technical, and narrowly construed policy issues well. However, it is not designed to address broad societal questions in which entrenched interests have divergent agendas. That is a job for real politics, not the lower tier of political sensitivity that scientists can reasonably be expected to master. In our defense, the intellectual property issues surrounding genomics overwhelmed even the big-league political system in the United States. The U.S. Patent Office went in one direction, federal agencies such as the NIH adopted inconsistent policies, and the biotechnology industry strongly favored gene patenting while major pharmaceutical companies were more comfortable with the “public trust” idea. In the end, a huge number of patents on gene sequences issued but few of them proved valuable. In 2013, a full 25 years after the *Mapping and Sequencing* report issued, the United States Supreme Court unanimously rejected the patentability of human genes. Many scientists, including me, welcomed the Supreme Court decision but also found it incoherent. Hence, the issue will probably surface again. At least for now, it is settled law in the United States that genome sequences themselves, as opposed to cDNA sequences, annotations, and other value-added derivatives thereof, are the public trust that the NRC committee always wanted them to be.

On the whole, *Mapping and Sequencing the Human Genome* was a spectacular success. The NRC issues hundreds of reports a year. Most of them are scarcely read and have no perceptible influence on policy. However, the *Mapping and Sequencing* report unequivocally shaped subsequent events. The Human Genome Project evolved in the way it did, maintained political support in the United States, withstood an opportunistic challenge from the private sector, encompassed contributions from international collaborators, and achieved its goals. All this happened, in some significant part, because the report provided a workable, high-level framework within which the project could pursue its historic mission.

In 2011, 23 years after publication of the Mapping and Sequencing report, the NRC issued a report entitled *Toward Precision Medicine: Building a Knowledge Network for Biomedical Research and a New Taxonomy of Disease*. In basic respects, this report was a sequel to *Mapping and Sequencing*. The latter had promised that a human genome sequence would “greatly enhance progress in human biology and medicine”. By 2011, the sequence had been in hand for a decade, re-sequencing technology was improving explosively, and the broad outlines of how sequencing of individual genomes could enhance medical care were becoming clear. It was time to deliver on the original promise.

While most of what I have said about the *Mapping and Sequencing* report has been published elsewhere, can be readily corroborated by other people, or is at least familiar to many scientists, my comments on the *Precision Medicine* report are of a more preliminary and idiosyncratic nature. The report itself has received considerable notice, particularly since President Obama announced a major United States Precision Medicine Initiative in 2015. Certainly the name of the Obama initiative, and much of the rhetoric surrounding it, came from the NRC *Precision Medicine* report. However, the Precision Medicine Initiative has not yet launched in any substantial way, and it remains to be seen whether or not its broad contours will conform to the NRC report’s recommendations.

The idea for the NRC study that led to the *Precision Medicine* report took shape during discussions between David Walt, Alan Williamson, and myself in late October of 2009. We were all at a meeting of the Illumina Scientific Advisory Board near San Diego, California. Recognizing that large-scale sequencing of individual human genomes was going to become practical during the next few years, we were concerned that there was no clear plan to exploit this technological revolution to improve medicine. There was ample interest and activity but no clear plan. The situation reminded me of genomics in 1987. At that time, a growing number of scientists were beginning to analyze cellular genomes systematically, and to pursue increasingly ambitious mapping and sequencing projects. However, nearly all projects were on a small scale and were motivated by the local interests of particular laboratories. So, David, Alan, and I talked about ways in which sequences of individual genomes, in the years going forward, might be brought under an umbrella comparable to the Human Genome Project.

Alan, who had extensive experience in the pharmaceutical industry and had been a long-time advisor of the National Human Genome Research Institute, suggested that the right over-arching theme of such an initiative might be development of a new taxonomy of disease. In a few areas, cancer being the most notable example, genome sequencing already allowed finer classification of diseases that had previously been lumped together. One could readily imagine a large, long-range project to extend this success. Obviously, cancer is an easier case than most disease since cancer progression is driven by somatic mutations. Nonetheless, if DNA sequencing and ancillary analyses – for example, of gene-expression patterns and epigenetic changes – were applied to a large enough group of patients under treatment for the whole range of human diseases, perhaps major advances in disease taxonomy would be achievable in other areas of medicine, as well. Since diagnosis is the starting point for all medical treatment, Alan argued that a disease taxonomy, broadly and deeply rooted in molecular pathology, would provide the most promising path toward leveraging the rapidly improving technology of genome sequences to achieve improved health outcomes in mainstream medicine.

David Walt and I signed on to Alan’s idea and began to discuss, while we were still in San Diego, how to act on it. Alan was thinking in terms of advocating NIH funding of pilot projects, but I argued that the idea was too big for that mechanism. Because of my experience with the NRC – not just my service on the Mapping and Sequencing Committee, but subsequent involvement as chair of a 2005 committee that issued a report entitled *Mathematics and 21st Century Biology* – I thought the new-taxonomy idea would benefit from an NRC study. The great advantage of these studies is that they can, at their best, give clear definition to otherwise vague ideas, provide a roadmap forward, and help launch subsequent discussions within the federal agencies where implementation decisions will ultimately be made. David and Alan went along with my suggestion, and the three of us agreed to approach Francis Collins, the NIH Director who we all knew well, about the possibility of obtaining NIH funding for an NRC report that would explore the potential use of large-scale molecular data to develop a new taxonomy of disease.

During the next few months, we pursued this goal through E-mail correspondence and conference calls among ourselves, with Collins, and with his staff. Collins proved supportive, although he had misgivings about turning the study over to the NRC rather than doing it in house. Although NRC studies are not cheap, typical budgets range from a half million to one million U.S. dollars, the NIH Director has sufficient discretionary funds to act quickly on initiatives of this type if he decides they are in the agency’s interest. After Alan, Walt, and my initiative in late 2009 and early 2010, discussions moved to venues within the NIH and NRC to which I had no access. However, they obviously went well. By March, 2010, we had feedback from both organizations indicating a study was likely to be launched. In October, committee recruitment was underway, and an NRC staff member asked me if I would agree to be nominated for the committee. I agreed and was ultimately appointed. The first meeting occurred in mid-December of 2010.

Before discussing the committee’s work, I would like to comment on the obvious conflict of interest issue posed by my service on both the Illumina Scientific Advisory Board (SAB) and the Precision Medicine Committee. As the leading manufacturer of DNA-sequencing instruments and reagents, Illumina would obviously benefit from a major, federally funded project to increase applications of DNA sequencing to medicine. First, this conflict is less substantial that it might appear. Illumina paid me a few thousand U.S. dollars to attend an annual meeting of the SAB. I had a year-to-year contract, no management responsibility, and owned no stock in the company. I had little access to proprietary information and what little I had was covered by a strong non-disclosure agreement. Second, the main way that conflicts of interest are handled within the scientific community in the United States is through full disclosure. Then, it is up to institutions such as universities, the NIH and the NRC to decide what action to take. They may decide that a particular conflict is inconsequential, that it is significant but should not disqualify a scientist from being an adviser, or that is disqualifying. Right at the beginning of my discussions both with Francis Collins and the NRC I disclosed my service on the Illumina SAB. Collins chose simply to take it into account while hearing out what David Walt, Alan Williamson, and I had to say. The NRC carried out a full review and decided the conflict was inconsequential. Nonetheless, at the first meeting of the committee, I brought it up so that other committee members could make their own judgments as to whether I seemed biased, as committee deliberations proceeded, by my relationship to Illumina. These issues are taken with the utmost seriousness in the United States. Everyone recognizes that public trust in science is our most valuable asset and is easily undermined by careless handling of conflicts of interest. The conflicts themselves are ubiquitous. That is actually a good thing. Universities, research institutes, government, and the private sector need to work closely together to achieve public benefit from scientific advances. It serves no one’s interest to partition the modest number of people who are expert in emerging areas of science into non-interacting sub-groups.

The Precision Medicine Committee met four times in late 2010 and the first half of 2011. During the spring of 2011, I and many other committee members put extensive time into the actual writing of the report. There were some conference calls and extensive e-mail exchanges, but no face-to-face meetings, during the period of most intensive report-writing. We had support from a professional writer, but he limited his input to light editing of the draft report for stylistic consistency. The final report was written almost entirely by committee members.

The most striking feature about the committee’s deliberations was the way in which the committee expanded its mandate as it went along. The Statement of Task, published in the report Appendix, does not say anything about precision medicine. It does not even say much about medicine. Our mandate was to “explore the feasibility and need, and develop a potential framework for creating a ‘New Taxonomy’ of human diseases based on molecular biology”. This wording hues closely to Alan Williamson’s initial concept. The Statement of Task for an NRC report is a carefully worded document that is negotiated between the sponsor for a report, the NIH in our case, and the NRC. Once set, it is virtually impossible to modify, and the NRC staff is charged with seeing to it that the committee sticks to it. We stuck closely enough to pass NRC review, but strained at the leash throughout the process.

There were several reasons we found ourselves unwilling to cling too tightly to taxonomy as a theme. A central one was our recognition that research and clinical application were going to have to co-evolve over a period of decades before molecular data could reasonably be expected to have major effects on mainstream medicine. We found ourselves wanting to recommend a process rather than a project. The process would be a gradual reorganization of the way we study human genotypes, other biomarkers, and phenotypes. The goal of this reorganization is schematicized in Figure 1–3 of the report. This figure was drawn and re-drawn several times. It became the centerpiece of the final phase of committee discussions. The schema’s most salient feature is a continuously updated “information commons”, indexed to individual patients. This idea may sound obvious, but, when examined closely, it reveals its radical stripes. I will highlight just a few reasons why it is a radical proposal:

1. The commons must be a commons. This may prove to be the deal-breaker for the committee’s vision. Everyone nods when anyone advocates widespread access to data. We are back to the public-trust issue of the *Mapping and Sequencing* report. However, the reality is that a dense thicket of national, institutional, commercial, and academic interests all militate against building a commons. Without aggressive push-back from policy-makers, many of whom have their own reasons to prefer keeping data bottled up, we will end up with a system so Balkanized that it will serve no one’s interests well.
2. The commons must include rich phenotypic profiles of individuals. The most important phenotypes for precision medicine are those affecting their health. If we are talking about millions of people, the only conceivable source of these data is the health-care system itself. There are few, if any, countries that have health-care systems organized in a way that will facilitate collection of these data. The system in the United States is particularly inimical to this goal.
3. The commons must be continuously updated. People’s health changes. Standards of care change. Molecular techniques change. Our primary interest is in health outcomes following medical interventions. In aging populations with a high burden of chronic disease, outcomes only become apparent over a period of years or decades. Only a process tightly integrated with the health-care system has any chance of tracking individuals over these periods. In mobile, modern societies, we do a poor job tracking even the most basic information about individuals – for example, their vaccination records – from one year to the next.
4. The commons must be indexed to individual patients. This point may seem obvious, but there is not presently a single person in the world whose molecular and phenotypic data I, a scientist qualified to participate in precision-medicine research and interested in doing so, can analyze in the comprehensive way envisioned for all patients in the information commons. Many vested interests do not want this situation to change. Patients would benefit if it did, but most researchers, health-care organizations, and government officials would not. Patients rarely win when confronting these powerful interests, particularly when few patients are well informed about the issues.

The Human Genome Project could set up shop, achieve its goals, and celebrate success. A Precision Medicine Initiative cannot proceed in this way. The goal is more effective use of molecular data in medical care. The ultimate deliverable is better health at affordable costs. These are not goals that can be achieved once and for all. Success will just increase demand for more success. Hence, the Precision Medicine Committee found itself recommending creation of an open-ended process, not pursuit of a bounded project. The *Precision Medicine* report should be read as a vision document. The committee saw it in that way.

Toward the end of our deliberations, we confronted the question of what title to give our vision document. “Taxonomy” seemed too big and technical a word to highlight. Furthermore, with our process- rather than project-oriented view of what needed to be done, we saw improved diagnoses as just one component of our vision. We were really talking about an effort to integrate basic research, translational research, clinical research, and the actual practice of medicine on a scale that had never before been attempted. We knew we wanted to work toward something big, but we did not know what to call it. In a moment of discouragement during our brain-storming, Steve Galli, the Chair of Pathology at the Stanford University School of Medicine, spoke up. Galli had not been a highly vocal member of the committee, but everyone knew he thought clearly about the big issues. Hence, whenever he joined the discussion, we all listened. Galli calmly suggested “*Toward Precision Medicine*” as a title for the report. At first, this idea took the rest of us by surprise. The phrase “precision medicine” had never come up during our months of discussion. No one liked “personalized medicine”, the common label for tailoring medical treatments to an individual’s genotype and molecular profiles, but, until Galli spoke up, no one had a better idea. He quickly won us over.

“Precision medicine” was the right choice and, in the long run, popularization of this phrase is likely to be the most lasting legacy of the committee’s work. The problem with “personalized medicine” is that, if taken seriously, it pushes medicine back toward the bad old days when physicians based too much of what they did on anecdotal experience and what little was then understood about human physiology. Most of medicine’s persistent follies arose because of overreactions to apparent successes in treating individual patients or use of interventions that seemed logical based on first principles, not actual experience. Without ever being tested in properly controlled trials, interventions spread into standard practice. The main reason that medical care today is immensely more effective than it was 50 years ago is that the medical community learned to reject this approach. “Evidence-based medicine” became the rallying cry for young physicians. For medicine to be solidly based in science, homogeneous groups of patients must be identified and some treated one way and the rest another. Nothing else works. Particularly dangerous is reliance on first principles to choose untested treatments. We are centuries away from understanding human biology well enough to choose interventions in this way. If the *Precision Medicine* report does nothing more than prod people to recognize these key points, something that is already happening, it will make a valuable contribution toward a healthier future for us all.
